# Cross-cultural and factorial validity of PTSD check list—military version (PCL-M) in Sinhalese language

**DOI:** 10.3402/ejpt.v4i0.19707

**Published:** 2013-02-12

**Authors:** Saveen N. Semage, Sivagurunadan Sivayogan, David Forbes, Meaghan O'Donnell, Roshan M. M. Monaragala, Emma Lockwood, David Dunt

**Affiliations:** 1 Department of Public Health, Sri Lanka Army Medical Services, Colombo, Sri Lanka; 2 Centre for Health Policy, Program and Economics, Melbourne School of Population Health, University of Melbourne, Australia; 3 Faculty of Medical Sciences, University of Sri Jayawardenepura, Sri Lanka; 4 Australian Centre for Post Traumatic Mental Health, University of Melbourne, Australia

**Keywords:** PTSD, PCL-M, Sri Lanka Army, cross-cultural validation, factorial validity

## Abstract

**Background:**

There are currently no validated instruments to assess the burden of combat-related Posttraumatic Stress Disorder (PTSD) in Sinhalese—the main spoken language in Sri Lanka.

**Objective:**

The purpose of this research was to establish the cross-cultural and structural validity of the PTSD Check List—Military Version (PCL-M) translated into Sinhalese.

**Methods:**

Expert committee consensus generation as well as translation–back translation approaches were used to establish the semantic, conceptual, and content equivalence of the Sinhalese and English versions of the PCL-M. Four translations of each item were made. In the absence of any “gold standard” psychometric instrument in Sinhalese to establish the criterion validity for the PCL-M (SIN), the study utilized more informal checks for assessment of validity and Sri Lankan cutoffs for caseness for PTSD to establish the psychometric strength of the translated instrument along with standard reliability analysis.

Confirmatory factor analysis was performed on PCL-M scoring of a random sample of 1,586 soldiers to examine construct validity.

**Results:**

Thirteen of the 17 items were selected by popular vote, and the remaining 4 through discussion and consensus. Reliability measured by Cronbach's-α was 0.944 for the total scale and 0.812, 0.869, and 0.895 for the three DSM-IV sub-scales (re-experiencing, avoidance/numbing, and hyperarousal), respectively. The desired cutoff point for the translated instrument was determined to be 44.

The five-factor model by Elhai et al. and the four-factor model by King et al. fitted best, demonstrating good fit to all three fit indices, while the four-factor model and the DSM-IV three-factor model by Simms et al. only had acceptable levels of fit for root mean squared error of approximation. χ^2^ difference test comparing the two better-fitting models suggests that the five-factor model by Elhai et al. has the better fit.

**Conclusion:**

The PCL-M (SIN) version is suitable for use in the study of PTSD in the Sri Lankan military forces, as judged by cross-cultural and construct validity as well as reliability.

Posttraumatic Stress Disorder (PTSD) is a psychiatric disorder that has been associated with trauma exposure, including combat exposure. High prevalence of PTSD among combat soldiers has been reported in many countries (Long, MacDonald & Chamberlain, 1996; Ramchand et al., 2010). Sri Lanka has experienced a very costly civil war over three decades, resulting in the deaths of tens of thousands of people while much larger numbers have been left physically or mentally disabled (South Asia Terrorism Portal 2012). Therefore, while a large number of PTSD cases could be expected from Sri Lanka, there is a dearth of research on the prevalence of combat-related PTSD in Sri Lanka and in the Asian region in general. To the best of our knowledge, the only study conducted in the Sri Lanka Army was a hospital-clinic-based study. It reported a prevalence rate of 6.7% (Fernando & Jayathunge, 2004). A critical factor limiting the conduct of research assessing the burden of PTSD is the absence of a validated screening instrument in the native language.

There are many reasons as to why standardized and validated research instruments should be used to estimate prevalence rates. Validated instruments enable the comparison of results across different countries and cultures as well as increase the confidence that they accurately reflect what they are supposed to measure (Herdman, Fox-Rushby & Badia, 1998). It is suggested that prevalence studies should always include a validation substudy—a recommendation which is often neglected (Michel et al., 2007). If a comprehensive adaptation process has not been undertaken to achieve validity of a cross culturally translated instrument, applying cutoffs or diagnostic algorithms may lead to misclassification and distort prevalence estimates (Canino & Alegria, 2008). Although there is no universal agreement as to how best to conduct cross-cultural translation in another cultural setting, there is agreement that it is inappropriate to simply translate and use a questionnaire in another linguistic context (Gjersing, Caplehorn & Clausen, 2010; Herdman et al., 1998). Despite this, a review on translations of psychiatric rating scales in the Southeast Asian region revealed that out of 13 translated scales, the majority had used a simple translation process (Ahmer, Faruqui & Aijaz, 2007). More rigorous studies use expert committee consensus generation in addition to conventional translation–back translation method to achieve consensual validation in cross-cultural validation process (Gjersing et al., 2010; Kohrt et al., 2011; Sumathipala & Murray, 2000). For example, Sumathipala and Murray (2000) used an expert committee for consensus generation to develop a culturally appropriate Sinhalese translation of the Bradford Somatic Inventory. It should be noted that in this study, the same expert committee was used for translation as well as consensus generation. Kohrt et al. (2011) added a further step in validating the Depression Self-Rating Scale and Child PTSD Symptom scale in Nepalese by employing focus group discussion with Nepali children to evaluate the translations done by the expert committee. As a different method of cross-cultural validation, Orlando and Marshall (2002) used two samples of English- and Spanish-speaking trauma survivors to attest the semantic equivalence of Spanish version of PTSD Check List (PCL) in addition to double translation procedures.

Criterion validation requires a different process. The usual process is for non-English versions of well-established instruments to be rated against an existing gold standard. However, in situations where these instruments have not been translated to the language of reference, the next available option is to assess the diagnostic properties of the instrument, including diagnostic cutoff points and sensitivity and specificity, using clinical diagnosis based on standard structured interviews.

Demonstrating that a translated instrument exhibits a similar latent structure to that of the original version further enhances its validity by means of construct validity. However, in terms of PTSD, even for the original version of PTSD Check List—Military Version (PCL-M), there is little support for the three-factor model of re-experiencing, avoidance/numbing, and hyperarousal described in DSM-IV (American Psychiatric Association, 2000), and there is no clear consensus regarding an alternative model (Palmieri, Weathers, Difede & King, 2007). The two most empirically supported models of the latent structure of PTSD symptoms were developed by King et al. and Simms et al. (Elhai et al., 2011), both of which are four-factor models. The emotional numbing model by King et al. separated DSM IV's avoidance/numbing cluster into separate clusters (King, Leskin, King & Weathers, 1998). The dysphoria model by Simms et al. moved sleep disturbance, irritability, and impaired concentration symptoms from hyperarousal to emotional numbing symptoms to create a novel factor called general dysphoria or distress (Simms, Watson & Doebbeling, 2002). A meta-analytic investigation of 40 PTSD studies found that the model by Simms et al. fitted best regardless of the measure used and the sample type, while both models showed good fit across subsamples of studies (Yufik & Simms, 2010). Elhai et al. (2011) demonstrated that a five-factor model in which sleep disturbances, irritability, and impaired concentration were separated into a new factor fitted their data better than two four-factor models. Among few instances where structural validity of PCL was tested in non-English speaking cultures, Wang et al. (2011) demonstrated a better fit for the model by Elhai et al. over other models using the Chinese version.

The aim of this study was to validate a PTSD scale in the Sinhalese language by 1) adopting a comprehensive cross-cultural translation process, using translation–back translation and expert committee consensus generation, 2) conducting best available alternative criterion validation process to test the instrument's utility in members of Sri Lankan military, using clinical diagnosis based on standard structured interviews, and 3) examining its latent structure through confirmatory factor analysis (CFA). The PCL-M (Weathers, Litz, Herman, Huska & Keane, 1993) was chosen as the instrument to use because it is a short, simple, self-report measure of PTSD symptom severity that meets Watson's five criteria (Watson, 1990).

## Methods

Ethical clearance for the research was obtained from the Ethical Review Committee of the Faculty of Medical Sciences of University of Sri Jayawardenepura, Sri Lanka.

### Cross-cultural translation

The PCL-M was first translated into Sinhalese separately by four different professionals, fluent in English and Sinhalese. The professionals included a military psychiatrist, a graduate English teacher, a community medicine registrar, and a lawyer who was frequently involved in the translation of documents. Before the translation, they were briefed about the objectives of the translation process and were asked to pay special attention to the cultural appropriateness and simplicity of wordings. Then in a group setting, all four translations were assessed by seven professionals from the fields of public health, psychiatry, military medicine, and English language. The panel was briefed about and provided with the English version of the instrument, the four Sinhala translations, and a list of symptoms elicited by each item of the instruments. The panel voted for the preferred version of each item based on expression of symptom, simplicity, and cultural appropriateness, and worked collectively to produce the final translation.

The translated instrument was later pretested among a group of 10 soldiers who attended the annual medical examination at the Military Hospital, Colombo, to assess the time taken to complete as well as its comprehensibility. Two psychiatrists who possess a long-time experience in treating combat-related PTSD patients further assessed the translation for face and content validity.

### Assessment of validity, reliability, and Sri Lankan cutoffs for caseness for PTSD

Reliability of a composite scale was measured by appraising internal consistency (Abramson & Abramson, 1999). Internal consistency was assessed by calculating the Cronbach's-α coefficient (Cronbach & Meehl, 1955). Internal consistency estimates of a magnitude of 0.70 or greater are considered satisfactory (Nunnally & Bernstein, 1994).

It was not possible to compare the psychometric properties of the PCL-M (SIN) using a comprehensive psychometric instrument, such as Clinician-Administered PTSD Scale (CAPS) or the World Mental Health Composite International Diagnostic Interview (CIDI) (Blake et al., 1995; Kessler & Ustun, 2004), as “gold standard” because these instruments have not been translated and validated in Sinhalese. Consequently, more informal checks were made as follows.

Limited assessment of validity and Sri Lankan cutoffs for caseness for PTSD were made by comparing results generated for the PCL-M and the clinical diagnosis of PTSD in 100 military personnel, 49 of whom had a previous clinical diagnosis of PTSD while 51 did not. This comparison was made at the soldier's annual medical examination after obtaining written informed consent. The PCL-M (SIN) was completed by the military personnel who were also clinically assessed for PTSD. The clinical assessment of PTSD status was conducted by two military psychiatrists of Military Hospital, Colombo, who were blind to the diagnostic status of the personnel. The psychiatrists were instructed to use the guidelines of CAPS (Blake et al., 1995) for clinical diagnosis. The “PTSD” and “non-PTSD” cutoff points to PCL-M-SIN were determined based on the receiver-operated characteristics (ROC) curve using the data of the validation study.

### Construct validity

A random sample of 1,586 male, currently serving soldiers of Sri Lanka Army was selected using multistage random sampling, and PCL-M Sinhalese version was self-administered. Assessment of fit of the three-factor DSM-IV model, the four-factor models by King et al. and Simms et al., and five-factor model by Elhai et al. was conducted with CFA. A one-factor model, which subsumes all 17 PTSD symptoms under a single general-factor model, and a two-factor model with intrusions/avoidance and numbing/hyperarousal, forming two separate factors were also included in analysis for comparison purposes (item allocations shown in Table 1).

**Table 1:** Item mapping for hypothesized factor models

	Model					
	
DSM-IV PTSD symptoms	Single factor	Two factor	DSM-IV	King	Simms	Elhai
B1.Intrusive thought	P	I,A	I	I	I	I
B2.Recurrent dreams	P	I.A	I	I	I	I
B3.Flashbacks	P	I,A	I	I	I	I
B4.Emotional reactivity	P	I,A	I	I	I	I
B5.Intrusive thought	P	I,A	I	I	I	I
C1.Avoiding thoughts	P	I,A	A,N	A	A	A
C2.Avoiding reminders	P	I,A	A,N	A	A	A
C3.Inability to recall	P	I,A	A,N	N	D	N
C4.Loss of interest	P	H,N	A,N	N	D	N
C5.Detachment	P	H,N	A,N	N	D	N
C6.Restricted affect	P	H,N	A,N	N	D	N
C7.Foreshortened future	P	H,N	A,N	N	D	N
D1.Sleep disturbance	P	H,N	H	H	D	X
D2.Irritability	P	H,N	H	H	D	X
D3.Difficulty in concentrating	P	H,N	H	H	D	X
D4.Hypervigilance	P	H,N	H	H	H	H
D5.Startling	P	H,N	H	H	H	H

Factors on which symptoms were loaded: P = general PTSD; I = intrusions; A = avoidance; N = numbing; D = dysphoria; X = separate factor for D1, D2, and D3 in Elhai model; H = Hyperarousal.

The variance–covariance matrix of the 17 PCL-M (SIN) item raw scores was the basis for each analysis, and robust weighted least squares (WLSMV) CFA was conducted using Mplus 6.1 (Muthen & Muthen, 1998–2010). To handle missing data, the default option was used, which involves listwise deletion of missing data. Goodness of fit was evaluated with χ^2^ statistics and other standard fit indices in line with the two-index strategy of Hu and Bentler (1998). This strategy places most emphasis on the root mean squared error of approximation (RMSEA) (Steiger, 1989), comparative fit index (CFI) (Bentler, 1990), and Tucker–Lewis Index (TLI) (Tucker & Lewis, 1973), as χ^2^ may be an overly sensitive test of fit in large samples (Cheng & Rensvold, 2002; Kline, 2010). An RMSEA of less than 0.06 and CFI or TLI greater than 0.95 is regarded as a good fit while an RMSEA less than 0.08 indicates an adequate fit (Hu & Bentler, 1998). Mplus version 6 also calculates 90% confidence intervals (CI) for RMSEA. WLSMV χ^2^ difference testing was used to directly compare the fit of well-fitting models, provided that those models were in a nested relationship (Brown, 2006).

## Results

### Cross-cultural translation

Table 2 shows the number of votes received for each item in four translations. Thirteen of the 17 items were decided by popular votes. The other four items (item no. 12, 15, 16, and 17) remained undecided after the round of voting. The 13 items for which consensus existed were reexamined by the panel, and a small number of changes in wording were made to strengthen the clarity and simplicity of the questions. The remaining four items were reworded collectively by the panel. A suitable Sinhalese phrase for “stressful military experience” was decided by the panel by general consensus, as that phrase is common for the first eight questions. In this way, agreement of the experts whether the underlying concept had been translated appropriately could be made.

**Table 2:** Preference for each item by the expert panel

Item no.	Translation 1	Translation 2	Translation 3	Translation 4
1	1	–	**4**	2
2	1	1	**5**	–
3	–	1	1	**5**
4	–	2	1	**4**
5	–	2	–	**5**
6	–	1	**6**	–
7	–	–	**7**	–
8	–	**4**	3	–
9	2	1	–	**4**
10	1	–	**4**	2
11	–	1	**5**	1
12	2	1	1	3
13	**4**	–	3	–
14	**4**	1	–	2
15	3	3	1	–
16	1	1	2	3
17	2	1	2	2

Bold values are the number of votes out of 7 expert panel votes.

### Assessment of reliability, validity, and Sri Lankan cutoffs for caseness for PTSD

#### Reliabilty

PCL-M Sinhala translation has an internal consistency of 0.944 while all the domains separately have shown an internal consistency of more than 0.8. Inter-item correlations were interpreted as suggested by Guiford in 1956 (Sprinthall, 1999). Item No. 3 showed a weak correlation with other items (item 1: 0.346, item 2: 0.363, and item 4: 0.348) except with item 5 (0.452). Item 8 showed low values with item 6 (0.263), item 7 (0.194), and item 12 (0.339). Item 12 showed low value with item 7 (0.339). All the other items showed favorable inter item correlations.

#### Validity and cutoffs for caseness

**Fig. 1:**
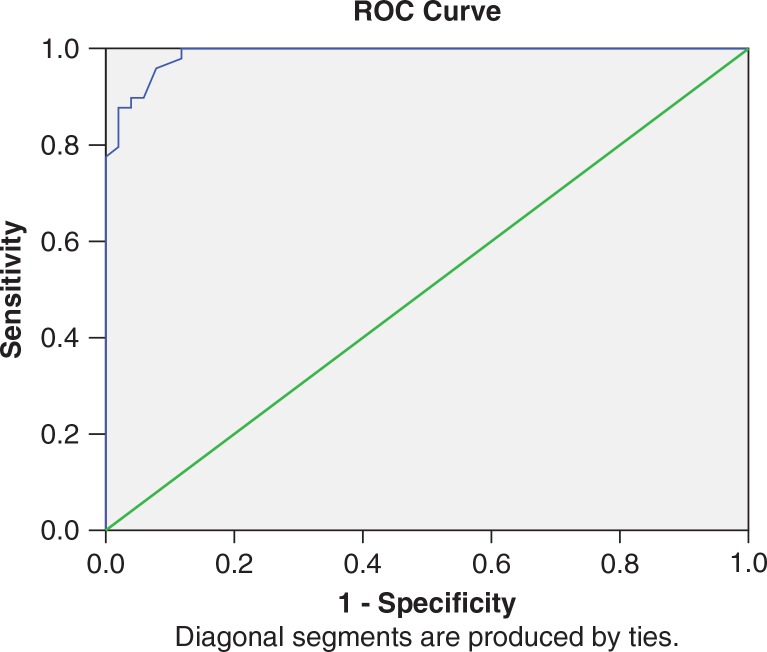
Receiver operator characteristic (ROC) curve of the PCL-M.

ROC curve (Fig. 1) and sensitivity and specificity values against each score show optimum balance at PCL-M cutoff score of 43.5. Area under the curve is 0.989 (95% CI: 0.976–1.002, *p*<0.000). Therefore, score of 44 was taken as the cutoff point for diagnosis of PTSD using Sinhala translation of PCL-M in the Sri Lankan military setting. With the decided cutoff point of 44, PCL-M Sinhala version reported a sensitivity of 95.9% (95% CI: 90.35–101.45) and a specificity of 92.2% (95% CI: 84.85–99.55). There is 100% sensitivity with the score of 39 and 100% specificity with the score of 56.

### Construct validity

Among our random study sample of 1,586 soldiers, 167 (10.5%, 95% CI: 9.02–12.04) met the diagnostic criteria for combat-related PTSD when the combined approach of cutoff point and DSM-IV diagnostic criteria was applied (Weathers et al., 1993). Baseline characteristics of the study sample are shown in Table 3. Fit indices for the hypothesized factor models are shown in Table 4. For all the models, χ^2^ values were significant relative to the degrees of freedom at *p*=0.001, indicating an absolute lack of fit. Thus, alternative fit indices were examined to evaluate the relative fit of the models.

**Table 3:** Sociodemographic characteristics and traumatic exposure

	No.	%
Age (years)		
18–25	792	50.1
25–35	567	35.9
More than 36	223	14.0
Rank		
Junior NCO	1,385	87.4
Senior NCO	142	9.0
Commissioned officer	57	3.6
Educational status		
Secondary education	1,450	91.8
Higher education	130	9.2
Marital status		
Unmarried	820	51.7
Married	750	47.5
Divorced/separated	8	0.5
Total military service		
Less than 5 years	854	54.1
5–15 years	518	32.7
More than 16 years	218	13.2
Special operational assignments		
Participated	434	27.5
Not participated	103	62.0
Age at first battle exposure		
18–20 years	841	53.5
20–25 years	618	39.3
More than 25 years	113	7.2
Traumatic exposure		
Battle injuries	496	31.6
Killing of rival combatants	1,162	73.3
Witnessing killing of rival combatants	1,349	85.1
Witnessing dead bodies	1,206	76.0
Childhood abuse	110	7.4

NCO = Non-commissioned officer.

**Table 4:** Goodness of fit indices for hypothesized models

Model	No. of factors	χ^2^	df	CFI	TLI	RMSEA (90% CI))
Elhai	5	472.478*	109	0.961	0.951	0.046 (0.042–0.050)
King	4	488.8918*	113	0.960	0.951	0.046 (0.042–0.050)
Simms	4	584.250*	113	0.949	0.939	0.051 (0.047–0.055)
DSM-IV	3	735.573*	116	0.933	0.922	0.058 (0.054–0.062)
Two factor	2	819.309*	118	0.925	0.913	0.061 (0.057–0.065)
Single factor	1	1395.552*	119	0.863	0.843	0.082 (0.078–0.086)

df = degrees of freedom; CFI = comparative fit index; TLI = Tucker-Lewis index; RMSEA = root mean square error of approximation; CI = confidence interval.

* Significant relative to degrees of freedom, *p*=.001.

Single-factor and two-factor models did not demonstrate goodness of fit according to any of the fit indices. The models by Elhai et al. and King et al. demonstrated good fit according to all three alternative fit indices while Simms’ and DSM-IV models demonstrated good fit according to RMSEA. RMSEA values for the models by King et al. and Elhai et al. fell outside the 90% CI for RMSEA for the model by Simms et al., suggesting that they fitted better than the model by Simms et al. WLSMV χ^2^ difference testing demonstrated that the model by Elhai et al. fitted significantly better than the model by King et al. (χ^2^=19.232, df = 4, *p*=0.0007).

## Discussion

Translation of the instrument was a crucial step in the validation process. According to Kleinman (1987), translation is the very essence of ethnographic research. Instruments are developed in a vernacular that may be quite difficult to translate into another language and strict lexical translations are often meaningless in non-Western cultures. Out of translation methods available for cross-cultural validation of screening instruments, “Expert committee consensus method” was preferred for this study over the simple translation–back translation method. The aim of the translation was to retain all the items in the original version and translate the items into culturally appropriate wording rather than word-to-word translation. Cross-cultural validation of Beck inventory, which demonstrated a new approach for translating instruments for cross-cultural research, used the same panel for translation and evaluation of those translations (Sumathipala & Murray, 2000). An additional step was taken in the present study by having separate expert panels for translation and evaluation, and modification of those translations.

### 

#### Defining cutoffs for caseness

The cutoff point for Sri Lankan setting (44) was found to be lower than original cutoff point of 50, suggested by the developers of the instrument (Weathers et al., 1993). Possible reasons for this change can be the cultural insensitivity to concepts, such as emotional numbing, the reluctance to accept detachment, and restricted affection to loved ones, again due to cultural bonding. This argument is well supported by the fact that the mean score for most of the items in cluster C (item no. 7–12) by PTSD patients had mean scores below the mean score of total items. The ROC curve analyses demonstrated that the PCL-M-SIN achieved a high level of diagnostic accuracy, in addition to solid reliability properties.

The CFA demonstrated that the latent structure of the translated instrument is broadly consistent with that of the English language version of the PCL-M, thus providing evidence for construct validity of the translated instrument. Out of the several factor structures empirically suggested for the original instrument, our study findings support the five-factor model by Elhai et al. as the best-fitting factor structure while the model by King et al. also demonstrated good and comparable fit. Among other studies using translated instruments, Wang et al. (2011) provide supporting evidence to our findings. The implications of using five-factor model in clinical setting needs to be further evaluated.

This study has several limitations. First, by selecting patients with well-defined PTSD symptoms and apparently mentally healthy people, the present study could not test the validity of the instrument to accurately diagnose patients with mild symptomatology or its discriminant validity with other psychiatric disorders. Second, the study used a hospital-based sample not a randomly selected sample from the general population. Therefore, only the two extremes of the disease spectrum are represented in the study sample. Third, our study used a military sample so that the generalizability of the findings to other trauma survivors is somewhat limited. Finally, assessment of PTSD symptoms using self-report instrument may affect the factor structure, as indicated by Palmieri et al. (2007).

## Conclusion

In conclusion, by adopting a stringent cross-cultural translation process and by demonstrating sound reliability and construct validity, Sinhalese translation of PCL-M can be considered as a valid and useful screening instrument for combat-related PTSD. Furthermore, our findings highlight the importance of adopting multidisciplinary expert committee consensus generation in cross-cultural translation of instruments. This study also found that the five-factor model by Elhai et al. provides the best-fitting factor structure for PTSD while the four-factor emotional numbing model by King et al. also demonstrates good fit. Therefore, the present study contributes to the limited literature regarding cross-cultural validation of PCL-M as well as latent structure of PTSD symptoms in non-English speaking cultures.
